# Supplementary material for: Evidence for multiferroicity in single-layer CuCrSe2
Source: Nat Commun. 2024 May 18;15:4252. doi: 10.1038/s41467-024-48636-z (PMC11102510; doi:10.1038/s41467-024-48636-z)
Supplement: Supplementary file 1 — Supplementary Information [file 41467_2024_48636_MOESM1_ESM.pdf]

Supplementary Materials for

Evidence for Multiferroicity in Single-Layer $CuCrSe_2$

Zhenyu Sun,[1,2,3#] Yueqi Su,[4,5,6#] Aomiao Zhi,[1,3,#] Zhicheng Gao,[1,3] Xu Han,[7] Kang Wu,[1,3] Lihong Bao,[1,3] Yuan Huang,[7] Youguo Shi,[1,3] Xuedong Bai,[1,3] Peng Cheng,[1,3] Lan Chen,[1,3,8*] Kehui Wu,[1,3,8,9] Xuezeng Tian,[1,3*] Changzheng Wu,[4,5,6*] Baojie Feng,[1,3,8,9*]

[1]*Institute of Physics, Chinese Academy of Sciences, Beijing, 100190, China*

[2]*Department of Chemistry, Brown University, Providence, RI 02912, USA*

[3]*School of Physical Sciences, University of Chinese Academy of Sciences, Beijing, 100049, China*

[4]*School of Chemistry and Materials Sciences, University of Science and Technology of China, Hefei, 230026, China*

[5]*CAS Center for Excellence in Nanoscience, and CAS Key Laboratory of Mechanical Behavior and Design of Materials, Hefei, 230026, China*

[6]*Collaborative Innovation Center of Chemistry for Energy Materials (iChEM), Hefei, 230026, China*

[7]*Advanced Research Institute of Multidisciplinary Science, Beijing Institute of Technology, Beijing, 100081, China*

[8]*Songshan Lake Materials Laboratory, Dongguan, Guangdong, 523808, China*

[9]*Interdisciplinary Institute of Light-Element Quantum Materials and Research Center for Light-Element Advanced Materials, Peking University, Beijing, 100871, China*

[#]These authors contributed equally to this work.

Figure S1 shows the X-ray diffraction (XRD) pattern of our initial $CuCrSe_2$ single crystal, where only the (00l) peaks are evident, and the experiemental diffraction positions are consistent with the theroretcial values. A picture of the crystals is shown in the inset. The XRD result suggests the high quality of $CuCrSe_2$ crystal, which is important for further chemical exfoliation.

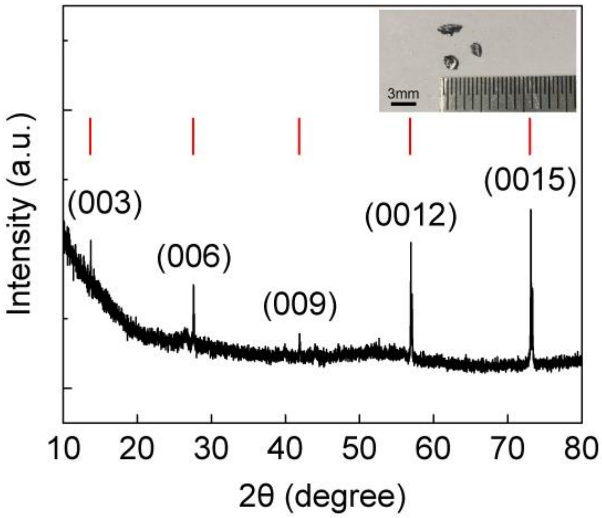

Figure S1. XRD pattern of the $CuCrSe_2$ crystal. Red lines indicate the calculated diffraction positions. The inset is an optical image of typical $CuCrSe_2$ crystals.

Figure S2 shows the Raman spectra acquired on $CuCrSe_2$ nanoflakes and silicon substrate.

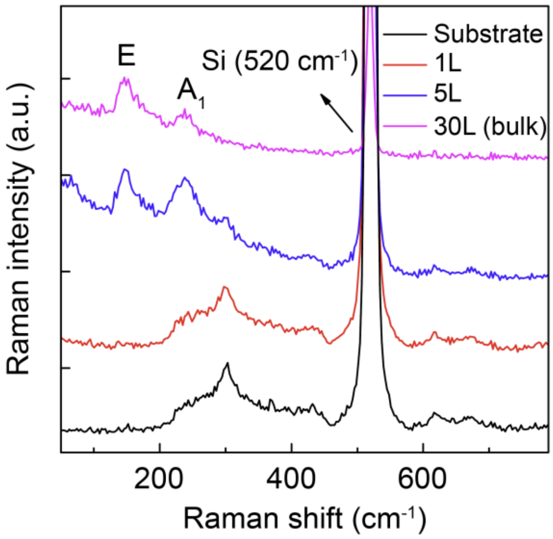

Figure S2. Raman spectra of the silicon substrate, 1L-$CuCrSe_2$, 5L-$CuCrSe_2$, and thick $CuCrSe_2$ nanoflakes, respectively.

Figure S3 demonstrates the clear 180° phase contrast between the two ferroelectric domains in Figure 2j after opposite bias poling. This characteristic can be served as a good indicator for the out-of-plane ferroelectricity in single-layer $CuCrSe_2$.

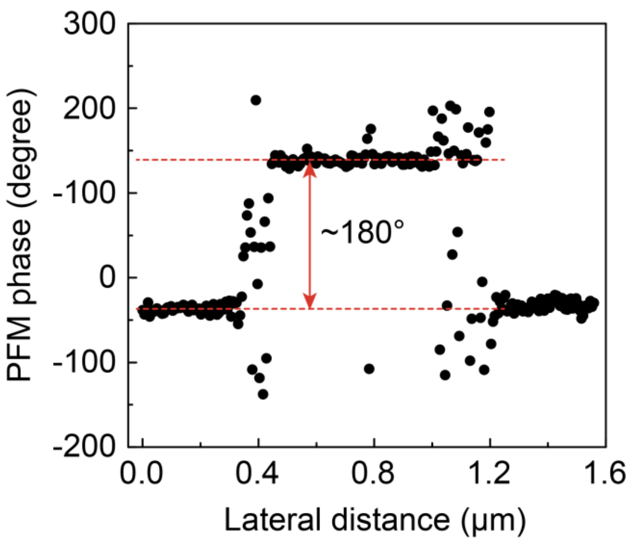

Figure S3. Line profile across the PFM phase image (Figure 2j) after domain writing.

Figure S4 indicates the good retain ability of the out-of-plane ferroelectricity in 1L-$CuCrSe_2$. Even after 7 days, the previously poled area can be still identified by PFM imaging.

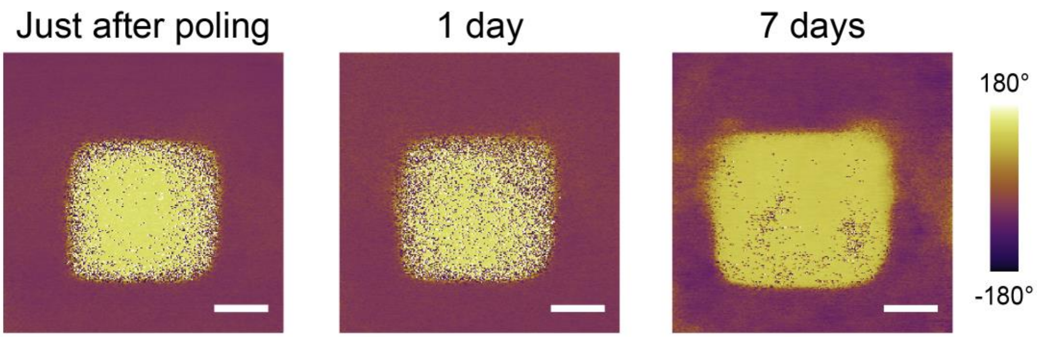

Figure S4. PFM phase image after opposite bias poling for different delay times.

Figure S5 displays the M-T curves of bulk $CuCrSe_2$. As the temperature decreases, the $CuCrSe_2$ bulk exhibits typical antiferromagnetic behavior with a T_N of 55 K, which is distinguished from the ferromagnetic behavior of $CuCrSe_2$ nanosheets.

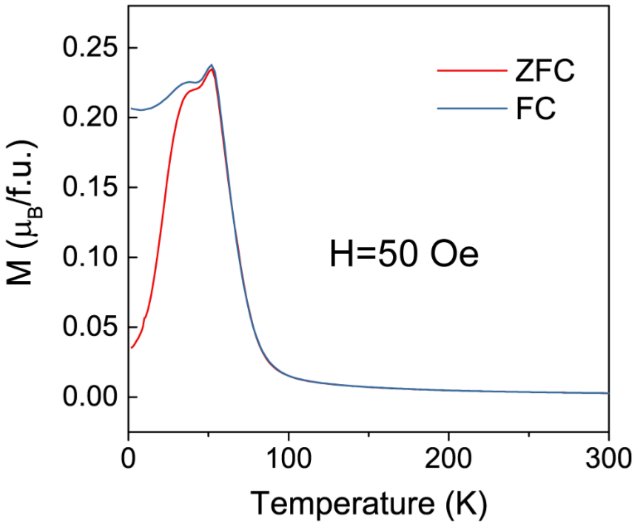

Figure S5. M-T curves of $CuCrSe_2$ bulk crystal.

Figure S6 shows the $CuCrSe_2$ nanosheets used for the macroscopic magnetic measurements.

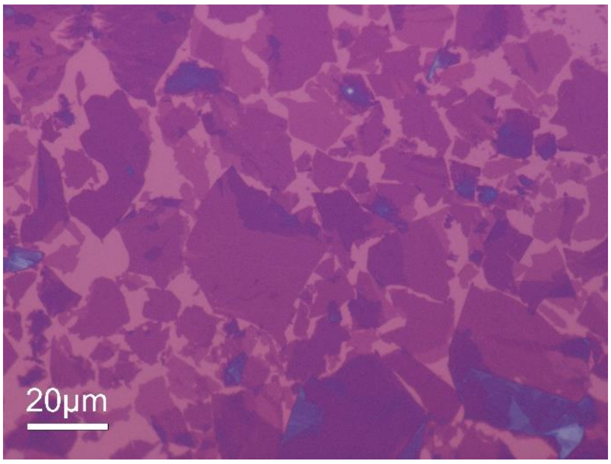

Figure S6. Typical optical image of the $CuCrSe_2$ nanosheets used for the magnetic measurement.

Figure S7 shows the characterization results of the 2L and 4L $CuCrSe_2$ sample used in our PFM and Hall measurements. All the nanosheets were fabricated by the redox-controlled chemical exfoliation.

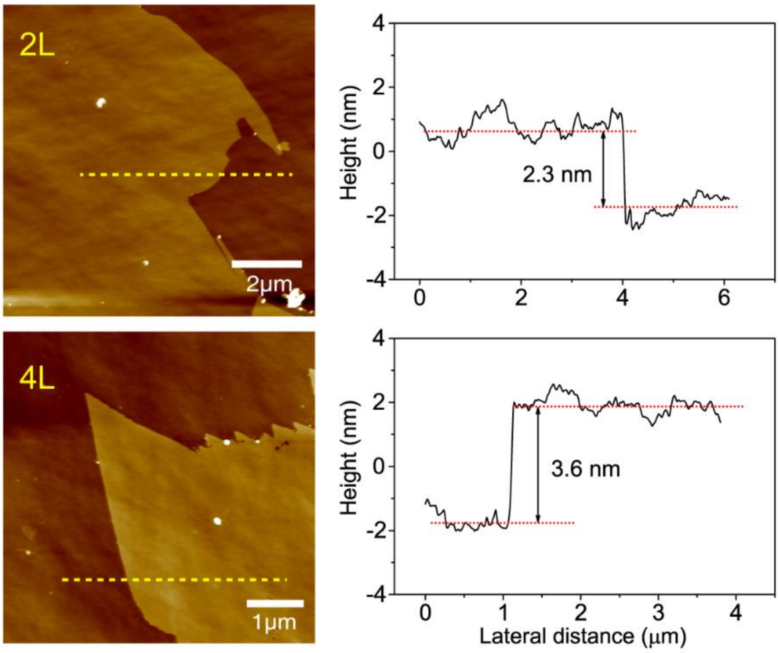

Figure S7. AFM image and height profile along the dashed line for 2L and 4L $CuCrSe_2$ sample.

Figure S8 shows the Hall response of a trilayer $CuCrSe_2$ device, here only the linear Hall effect can be observed.

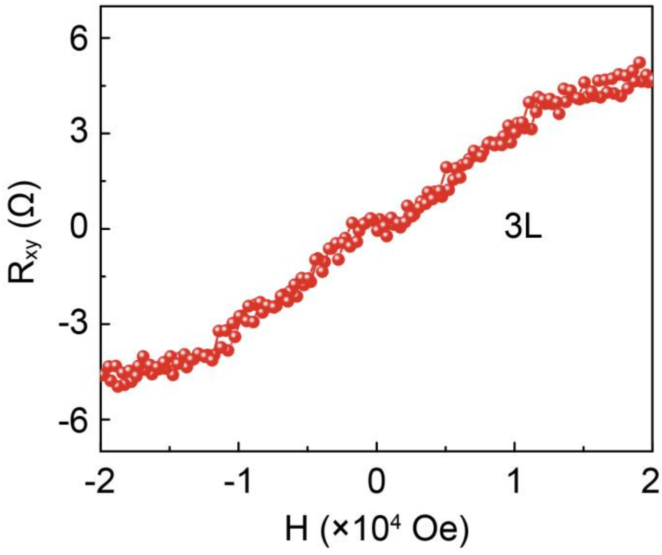

Figure S8. Out-of-plane magnetic field dependent Hall resistance for the 3L $CuCrSe_2$ sample, the measurement temperature was 2 K.

For PFM local switching spectroscopy measurement, a DC triangular voltage waveform is applied to the tip, meanwhile, a small AC signal is superimposed on it. A

typical DC waveform in our experiment is displayed in Figure S9a, where the PFM hysteresis loop is collected in the interval between two pulsed bias voltages (off-field state). Before each ferroelectric loop measurement, we need to ensure that the conductive PFM tip shows a great contact-resonance peak with our samples, as indicated in Fig. S9b.

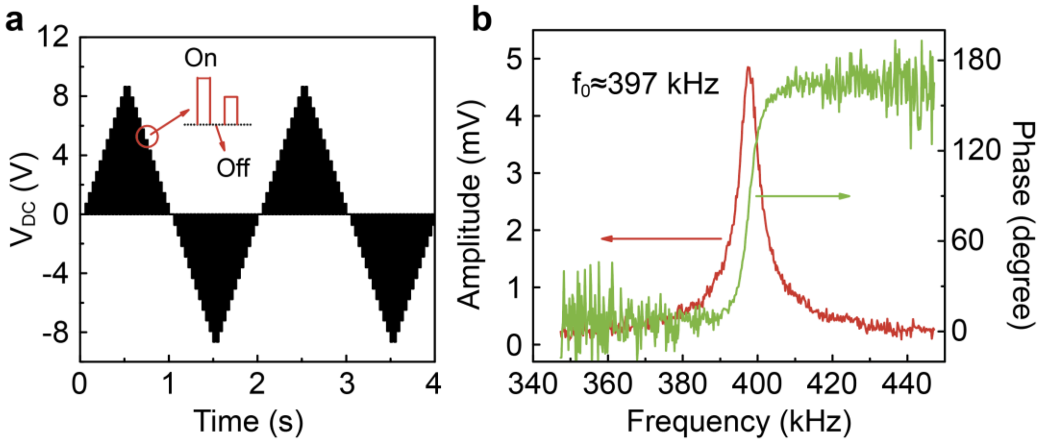

Figure S9. PFM local switching spectroscopy measurement in DART mode. (a) A typical DC waveform applied to the tip. The maximum bias is 9 V and the frequency is 0.5 Hz. (b) A representative contact-resonance peak before performing the PFM loop measurements.
